# Inducible ablation of CD11c^+^ cells to determine their role in skin wound repair

**DOI:** 10.1111/imm.13312

**Published:** 2021-03-01

**Authors:** Zhi Li, Rebecca Lamb, Mark C. Coles, Clare L. Bennett, Carrie A. Ambler

**Affiliations:** ^1^ Department of Biosciences Biophysical Sciences Institute Durham University Durham UK; ^2^ Department of Biology Centre for Immunology and Infection Hull York Medical School York UK; ^3^ Kennedy Institute of Rheumatology University of Oxford Oxford UK; ^4^ Institute of Immunity and Transplantation University College London London UK; ^5^ Division of Cancer Studies University College London London UK

**Keywords:** CD11c, dendritic cells, Langerhans cells, skin, wound repair

## Abstract

Whether resident and recruited myeloid cells may impair or aid healing of acute skin wounds remains a debated question. To begin to address this, we examined the importance of CD11c+ myeloid cells in the early activation of skin wound repair. We find that an absence of CD11c+ cells delays wound closure and epidermal proliferation, likely due to defects in the activation of the IL‐23‐IL‐22 axis that is required for wound healing.

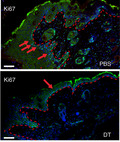

**Abbreviations**
cDC: conventional dendritic cell
DC: dendritic cell
dpw: days post‐wounding
DT: diphtheria toxin
DTR: diphtheria toxin receptor
ILC3: type 3 innate lymphoid cell
LC: Langerhans cell
mRNA: messenger ribonucleic acid
PBS: phosphate‐buffered saline

To the Editor,

Wound healing in healthy skin requires a complex interplay between immune and non‐immune cells. In addition to their roles in infection control and cell debridement, leukocytes secrete factors to orchestrate the timing of the repair process via crosstalk with epithelia. This critical role in wound timing is vital as failure to induce wound closure leads to debilitating chronic wounds susceptible to further infection and patient sepsis. We have shown that injury‐induced activation of Notch signalling results in recruitment of type 3 innate lymphoid cells (ILC3s) that control macrophage recruitment and epidermal closure.^1^ Injury also initiates the rapid influx of innate myeloid cells to the skin; this influx is initially dominated by neutrophils, followed by monocytes and dendritic cells (DCs).^2^ But whether innate myeloid cells help or hinder processes initiated by ILC3s is unclear. Moreover, resident myeloid cells such as epidermal Langerhans cells (LCs) will be destroyed upon tissue destruction. Whether and how these cells are important during the earliest activation of the wound healing response remains to be elucidated.

Focused on these questions, we read with interest the recent *Immunology* study by Rajesh et al.,^3^ in which they investigated skin wound healing in mice depleted of Langerin^+^ LCs and dermal conventional type 1 DCs (cDC1s), using the Langerin‐DTR (diphtheria toxin receptor) mouse. The authors demonstrated an initial acceleration in wound closure in skin lacking Langerin^+^ cells, which they suggested was consistent with the known negative or regulatory role for resident LCs in skin immunity.^4^ We have recently conducted a complementary study in which we ablated CD11c^+^ cells, including LCs, cDCs and other myeloid cells, in the CD11c‐DTR model to investigate the impact on closure of cutaneous wounds. In contrast to Ranjesh et al., we observed a marked delay in wound healing in the absence of these cells. We believe that comparison of these in vivo ablation studies, in which different but overlapping populations of cells were deleted before wounding, reveals novel insights into the role of myeloid cells in cutaneous wound repair that will be of interest to your readers.

## Methods and materials

#### Mice and wounds

CD11c.DTR mice (on the B6 background) were bought from the Jackson Laboratories and bred in‐house. Mice were wounded in accordance with methods published in Li et al. (2016) and Li et al. (2018).^1^, ^5^ Briefly, anaesthetized mice were given two full‐thickness, 4‐mm‐diameter back skin wounds with a minimum distance of 1 cm apart using a punch biopsy (Stiefel). Two days prior to and on the day of wounding, mice were injected intraperitoneally with two doses of 100 ng diphtheria toxin (DT, Sigma, D0564) or PBS. All experimental procedures were performed with ethical permission by Durham University and the University of York under UK Government Home Office licences to CA and MC (60/3941 and 60/4178). Experiments followed the national and institutional guidelines for the care and use of animals on the basis of the Animal (Scientific Procedures) Act 1986 and where possible ARRIVE guidelines.

Wounds were photographed using a 3D camera (Quantificare) on day 5 after injury. Each wound was photographed three times, with each image analysed three times to check for measurement consistency. A random code was assigned to each image, and blind analysis was carried out using the Dermapix software (Quantificare).

#### Flow cytometry

Quantification of immune cells within the skin wounds by flow cytometry was performed as previously described.^1^, ^5^ An 8 × 8 mm piece of mouse back skin including the wounded area and adjacent area was collected, and the ventral surface was scraped gently with a scalpel to remove subcutaneous fat and muscle. Tissue was cut into 4–5 smaller pieces and incubated with 0·35 mg/ml Liberase TL (Roche, 5401020001), 3 mg/ml Collagenase D (Roche, 11088866001) and 0·1 mg/ml DNase I (Roche, 10104159001) in a volume of 700 µl RPMI‐1640 (Life Technologies, 31870‐025) on a heating shaker at 37 °C for 2 h. After incubation, undigested debris was removed by filtering the sample through a 70‐µm strainer. Cells were harvested by centrifuging at 400 *g* at 4°C for 5 min and resuspended in FACS staining buffer (0·5% BSA, 2 mm EDTA in PBS). Following incubation with anti‐CD16/32 (BioLegend, 101320, 1:100) and rat IgG (Sigma, Cat: I8015‐10 mg; 1:1000) diluted in FACS staining buffer, cells were stained for viability dye eFluor 780 (Thermo Fisher, 65‐0865, 1:1500) and surface markers on ice for 30 minutes by using the following antibodies: CD11b PE‐Cy7 (BioLegend, M1/70, 101215, 1:1000), CD11c pacific blue (Thermo Fisher, N418, 117321, 1:200), CD45 PerCP‐Cy5·5 (BioLegend, 30‐F11, 103131, 1:100), CD207 PE (BioLegend, 4C7, 144203, 1:200) and F4/80 APC (eBioscience, BM8, 17‐4801, 1:400). Data were acquired on an LSR Fortessa (BD) and analysed with FlowJo and GraphPad Prism software.

#### Immunostaining

Tissue was collected and processed as previously described.^1^ 8‐μm‐thick frozen tissues were fixed in 4% paraformaldehyde, blocked in 10% goat or donkey serum, 0·25% fish skin gelatine and 0·2% bovine serum albumin and then stained with purified anti‐Ki67 antibody (Abcam, ab16667, 1:400) diluted in permeabilization buffer (Thermo Fisher, 00‐8333, 1:10) for 1 h. Tissue sections were stained with Alexa Fluor 488 secondary antibody (Thermo Fisher, A11008, 1:1000) and DAPI‐counterstained before imaging using a Leica Tandem SP5 confocal microscope. The brightness of images was adjusted using the ImageJ software.

#### RNA extraction and quantitative RT‐PCR

8 × 8 mm wounded back skin tissues were collected, and RNA was extracted from tissues immersed in RNAlater (Merck, R0901) for 24 h before freezing. Tissues were homogenized using Polytron tissue homogenizer, and then, RNA was isolated using RNeasy Mini Kit (Qiagen, 74104) following the manufacturer's instructions including the optional, on‐column DNase I digestion step using RNase‐free DNase set (Qiagen, 79254). An additional proteinase K digestion step was performed in the protocol: homogenized lysates were incubated with 0·2 mg/ml proteinase K (Merck, P2308) in RLT buffer (Qiagen) at 55°C for 10 min. Harvested RNAs were quantified using a NanoDrop microspectrophotometer, and cDNA was prepared using the High Capacity cDNA Transcription Kit (Thermo Fisher, 4368814).

FAM‐labelled TaqMan™ probes were used for IL‐22 (Mm01226722_g1; Thermo Fisher) and endogenous control GAPDH (Mm99999915_g1; Thermo Fisher). Triplicate reactions (20 μl) of each experimental sample were analysed using TaqManTM Fast Universal PCR Master Mix (Thermo Fisher, 4352042) and subjected to an initial 3‐min denaturation step at 95 °C, followed by 40 cycles of 95°C for 3 s and 60°C for 10 s.

Unlabelled primers were used for IL23a p19 (NM_031252·2, Forward 5′‐TGGAGCAACTTCACACCTCC‐3′, Reverse 5′‐GGCAGCTATGGCCAAAAAGG‐3′) and endogenous control beta‐2 microglobulin (B2M, NM_009735·3, Forward 5′‐GTCGCTTCAGTCGTCAGCAT‐3′, Reverse 5′‐TTTCAATGTGAGGCGGGTGG‐3′). Triplicate reactions (20 μl) of each experimental sample were analysed using SYBR Green JumpStart™ Taq ReadyMix™ (Merck, S1816) and subjected to an initial 3‐min denaturation step at 95 °C, followed by 40 cycles of 95°C for 10 s, 60°C for 10 s and 72°C for 10 s.

RT‐PCR was performed using a Rotor‐Gene Q instrument (Qiagen). Data were analysed using the comparative quantification algorithm in the Rotor‐Gene software, with calibrator samples for each run being compared in a common experiment.

## Results and discussion

**Figure 1:**
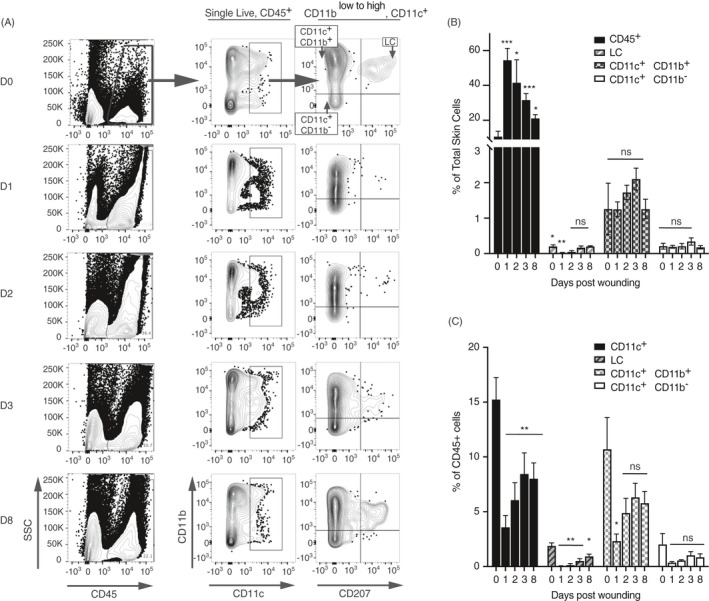
Flow cytometric analysis of CD11c+ populations in wounds. Mice were received 4‐mm full skin thickness wounds and the skin immunophenotyped at different time‐points. (A) Representative contour plots showing gated CD11c populations in wounded back skin at successive time‐points post‐wounding. Cells were pre‐gated on single live cells. Summary bar graphs show (B) the mean frequency ± SD of gated cells, calculated as a percentage of the total live skin cells or (C) of the CD45+ population for wounds 1, 2, 3 and 8 dpw. Averages are of three biological replicates. Each time‐point was compared with unwounded skin using the pairwise comparison *t*‐test with the Welsh correction. *P ≤ 0·05; **P ≤ 0·01; ***P ≤ 0·001; NS, not significant.

We examined the effects of acute damage on the kinetics of resident and recruited CD11c^+^ cells by flow cytometry, which allowed for precise cell quantification. Using methods detailed in our previously published work,^5^ cell populations were investigated in a 8 mm × 8 mm skin sample consisting of the wound bed and adjacent uninjured skin during the inflammatory stage at 1–3 days post‐wounding (dpw) and re‐epithelization at 8 dpw (Fig. 1A). Cutaneous wounding triggers a rapid transient influx and/or expansion of CD45^+^ immune cells at the damaged site; the percentage of CD45^+^ immune cells had more than trebled immediately after injury 1 dpw and remained high at 1–3 dpw compared with unwounded skin, but had returned towards baseline levels by day 8 dpw (Fig. 1B). Focusing on CD11c^+^ cells, we observed an early relative reduction in the frequency of CD11c^+^ cells as a proportion of CD45^+^ cells (Fig. 1B), in line with the rapid influx of neutrophils that occurs immediately after wounding.^2^ As the numbers of CD45^+^ immune cells declined post‐wounding, the proportion of CD11c^+^ cells increased, suggesting that these cells were becoming a more dominant part of the wound healing response from 3 dpw (Fig. 1C). Parsing of the CD11c^+^ population demonstrated that the majority of CD11c^+^ cells were CD11c^high^CD11b^int to high^ cells, which are expected to contain cDC2− and monocyte‐derived DC, although activated monocytes and macrophages were not formally excluded from this gate. CD11c^high^CD11b^int to high^ cells were diminished upon wounding but rapidly returned to baseline levels by 2 dpw, suggesting rapid recruitment to the wound bed. By comparison, we did not observe differences in the CD11c^high^CD11b^neg^ fraction (including Langerin^+^ cDC1) during the time course. Rajesh et al. quantified LC numbers by immunohistochemistry at and around the wound bed and demonstrated the destruction of resident LC followed by the apparent repopulation of the epidermis by 9 dpw.^3^ Our flow cytometric analysis also revealed the dramatic loss of LC on wounding, with gradual return of CD11b^int^CD207^+^ cells over time. However, we and others^2^ found LC frequency had not returned to baseline by 8 dpw (Fig. 1A‐C). Our studies and that of Rajesh et al. did not address the origin of the repopulating LC, which may be derived from dividing resident embryo‐derived LC and/or monocytes recruited to the inflamed site. However, it was notable that repopulation of LC was preceded by the increase in CD11c^high^CD11b^int to high^ cells in our study, which may contain monocytic LC precursors.^6^

**Figure 2:**
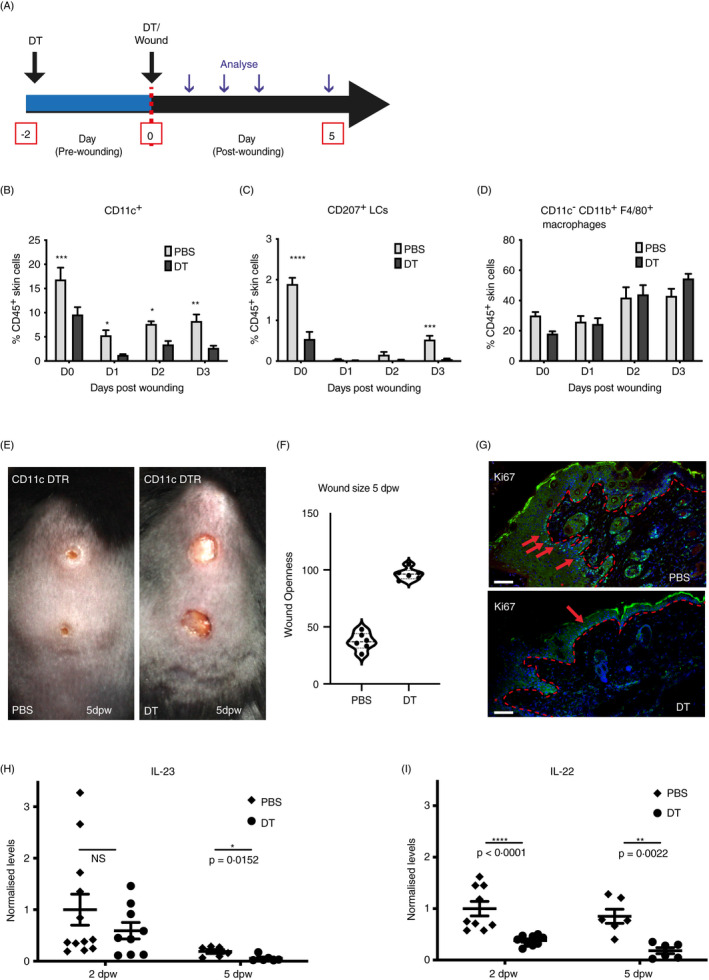
Ablation of CD11c+ cells delays wound closure and IL‐23/IL‐22 production in wounds. (A) Schematic showing the experimental protocol. CD11c‐DTR mice were injected with DT or PBS, as a control, 2 days prior to and on the day of wounding. (B‐D) Wounds were immunophenotyped by flow cytometry. Summary bar graphs show the mean frequency ± SD of all (B) CD11c^+^ cells (C), CD11b^int^CD207^+^ LCs and (D) CD11c^neg^CD11b^+^F4/80^+^ macrophages among total CD45^+^ cells, in unwounded (D0) and 1, 2 or 3 dpw in both DT‐treated and PBS‐treated CD11c‐DTR mice. Data are from three biological replicates. (E) Representative images of wounded PBS‐treated and DT‐treated CD11c‐DTR mice 5 dpw. (F) Wounds were photographed, and size was calculated as a percentage of the wound size on the day of wounding. Graphs show individual mice with lines at the mean. (G) Sections of wounds 5 dpw from PBS‐ and DT‐treated mice stained with an antibody to Ki67 (green) and costained with DAPI (blue). Red arrows indicate representative positive staining, and red asterisks mark epidermis adjacent to wound sites. Scale bars equal 100 microns. (H and I) qPCR quantification of IL‐23 and IL‐22 in total wound skin from PBS‐ and DT‐treated CD11c‐DTR mice analysed 2 and 5 dpw. Graphs show individual samples, with lines at mean ± SD, *n* = 9–12. Each time‐point was compared with unwounded skin (B‐D) or PBS‐treated control at 2 or 5 dpw (H and I) using the pairwise comparison *t*‐test with the Welsh correction. *P ≤ 0·05; **P ≤ 0·01; ***P ≤ 0·001; and ****P ≤ 0·0001; NS, not significant.

To determine the role of CD11c^+^ cells in wound closure, we used the CD11c‐DTR mouse model in which injection of diphtheria toxin (DT) results in the rapid inducible deletion of cells expressing a high‐affinity DTR.^7^, ^8^ CD11c‐DTR littermate mice were injected with DT or PBS, as vehicle control, 2 days prior to wounding and on the day of injury (Fig. 2A). This protocol resulted in the efficient and sustained deletion of CD11c^+^ CD11b^neg to high^ cells and CD11c^+^CD11b^int^CD207^+^ LC (Fig. 2B,C). There was no difference in the frequency of CD11c^neg^CD11b^+^ F4/80^+^ macrophages, which comprise a significant proportion of CD45^+^ cells at wound sites,^1^, ^2^ upon injection of DT (Fig. 2D).

To examine the impact of CD11c cell depletion on healing, wound size was measured in PBS‐ or DT‐injected mice for up to 5 days following injury, as previously described^5^ (Fig. 2E). Notably, wounds failed to re‐epithelialize and remained fully open in DT‐treated CD11c‐DTR mice (average openness 96·4%, *n* = 6) compared with PBS‐treated littermates (average openness 37·3%, *n* = 6) at 5 dpw (Fig. 2F). The re‐epithelialization phase of repair is marked by extensive proliferation in the epidermis at the wound edge and in the dermal fibroblasts.^9^, ^10^ In DT‐treated CD11c‐DTR mice, reduced proliferation in both compartments was detected by immunostaining with proliferation marker, Ki67, on sectioned wounds (Fig. 2G). This suggested that a reduction in cell proliferation contributed to the healing failure in these mice. Epidermal thickening at the wound edge was also reduced compared with PBS‐treated control wounds with only rare Ki67^+^ cells detected in the epidermis (Fig. 2G, arrows). Our and others’ work has shown that wound‐induced infiltration of macrophages plays key roles in the re‐epithelialization phase of tissue repair.^1^, ^11^ However as we did not detect changes in F4/80^+^ macrophages (Fig. 2D) our data suggested that loss of other CD11c^+^ cells, rather than a defective macrophage compartment, was the cause of aberrant wound closure observed in these mice. Together with our previous study,^1^ these findings imply non‐redundant roles for innate lymphoid and myeloid cells in cutaneous wound healing.

This absence of changes in macrophage frequency appears to differ from Rajesh et al., who detected an increase in the number of F4/80^+^ cells by immunohistochemistry 6 dpw.^3^ This difference may reflect the methodologies used to quantify cells, and different time‐points tested. However, it is also possible that broader depletion of all CD11c^+^ cells from our model, but not the Langerin‐DTR mouse, resulted in loss of precursors and/or cytokine‐producing cells that seeded the expanded F4/80^+^ pool in the skin. Neither study specifically considered the role of monocytes as either effector cells or precursors to other cells in the wound healing response. But, it is interesting to note that the increase in GM‐CSF upon deletion of Langerin^+^ cells observed by Rajesh et al.^3^ would directly impact on differentiation of monocytes into moDC in the dermis.

The decrease in the rate of wound closure upon loss of CD11c^+^ cells was in striking contrast to that of Rajesh et al. who demonstrated that injection of DT into Langerin‐DTR mice prior to, and after, wounding by excision of full‐thickness skin biopsies led to an initial rapid contraction of the wound.^3^ Subsequent closure in the latter study occurred at a similar rate to control animals, but the acceleration of healing within the first 24 h led to faster overall wound closure in skin depleted of Langerin^+^ cells. Thus, the absence of overlapping, but different, populations of myeloid cells appears to have a distinct and contrasting impact on the initiation of wound repair; whilst depletion of CD11c^+^ cells, including LCs, dermal cDCs and moDCs, in our study delayed wound closure, specific ablation of LC and Langerin^+^ cDC1 prior to wounding accelerated the healing response.^3^ The DT‐mediated absence of LCs from the skin before wounding in our study and that of Rajesh et al. (2020) may point towards the importance of LC in maintaining a regulated homeostatic immune environment in the absence of infection; indeed, LC numbers are decreased in non‐wounded skin of *db/db* diabetic mice that are susceptible to chronic wounding,^2^ and recent analysis of transcriptional networks within human LCs has revealed default pathways of tolerance in the absence of infectious signals.^12^ Subtle differences between these studies such as use of analgesia and animal cohousing conditions may impact outcomes; the use of antibiotics by Rajesh et al., but not in our study, will alter local cutaneous and systemic microbiota, which could alter immune cell dynamics and the rate of wound closure.^13^ It is also possible that, as we did not analyse the very early time‐points post‐wounding, LC‐dependent accelerated wound shrinkage within 24 h of wounding may also occur in our CD11c‐DTR model. However, we propose that the absence of recruited CD11c^+^ DCs at later time‐points overrides any impact of early loss of LC and ultimately dominates to delay healing.

Key roles for the IL‐23/IL‐22 signalling axis have been defined in facilitating immune–non‐immune cell crosstalk leading to wound closure.^9^, ^10^, ^11^, ^12^ To gain insight into the mechanisms by which CD11c^+^ cells may impact on wound repair, we examined the mRNA levels of IL‐23/IL‐22 in wounded CD11c‐DTR mice. IL‐23 mRNA levels were significantly lower in CD11c‐depleted wounds at 5 dpw (data at 2 dpw were not statistically significant) compared with normal wounds, whilst IL‐22 mRNA levels were lower at both 2 and 5 dpw in DT‐treated wounds. These results suggest that CD11c^+^ populations contribute to the earliest stages of skin repair, in part, through regulation of localized IL‐22/IL‐23 expression in skin wounds.

Influx of moDC/cDC2 to the wound site has been demonstrated by others^2^ and points towards a key role in healing. Whilst we did not specifically identify these cells, we also observed accumulation of CD11c^+^CD11b^+^ cells at the wound site. DT injection ablated CD11c^+^CD11b^+^ cells in our CD11c model, but they would have been present in DT‐injected Langerin‐DTR mice^3^ and may therefore contribute to the difference in our findings. Psoriasis may be viewed as aberrant activation of the wound closure response. Here, DC IL‐23 stimulates dermal Th17 cells to produce IL‐17A and, to a lesser extent, IL‐22, which drive the hallmarks of this pathology.^12^ We speculate that recruited moDCs/cDC2s contribute to the formation of the new epidermis; deletion of IL‐23‐producing moDCs/cDC2s in the CD11c‐DTR model prevents initiation of the IL‐22‐dependent proliferation and differentiation of keratinocytes, ultimately preventing the efficient healing of the acute wound. More detailed phenotyping of skin myeloid cells is required to test this hypothesis. However, in support for a key role for DC, there is evidence to suggest that a role for DCs could be conserved across different types of skin injury; depletion of CD11c^+^ cells in the CD11c‐DTR model also delayed healing of burn wounds, whilst injection of the growth factor FLt3L to increase DC numbers was sufficient to accelerate healing.^14^

In summary, whilst neither study provide the resolution to distinguish between the specific roles of LC and DC in wound healing, together they begin to shed light on the ways in which resident and recruited myeloid cells may impair or aid acute skin wound closure during the orchestrated process of healing. Defining these mechanisms has important clinical implications for improving the outcome of chronic wounds.

## Disclosure

The authors have no competing interests.

## Data Availability

The data sets generated during and/or analysed during the current study are available from the corresponding author on reasonable request.
